# Supplementary figures and images for: Selenium uptake, tolerance and reduction in Flammulina velutipes supplied with selenite
Source: PeerJ. 2016 May 11;4:e1993. doi: 10.7717/peerj.1993 (PMC4986802; doi:10.7717/peerj.1993)

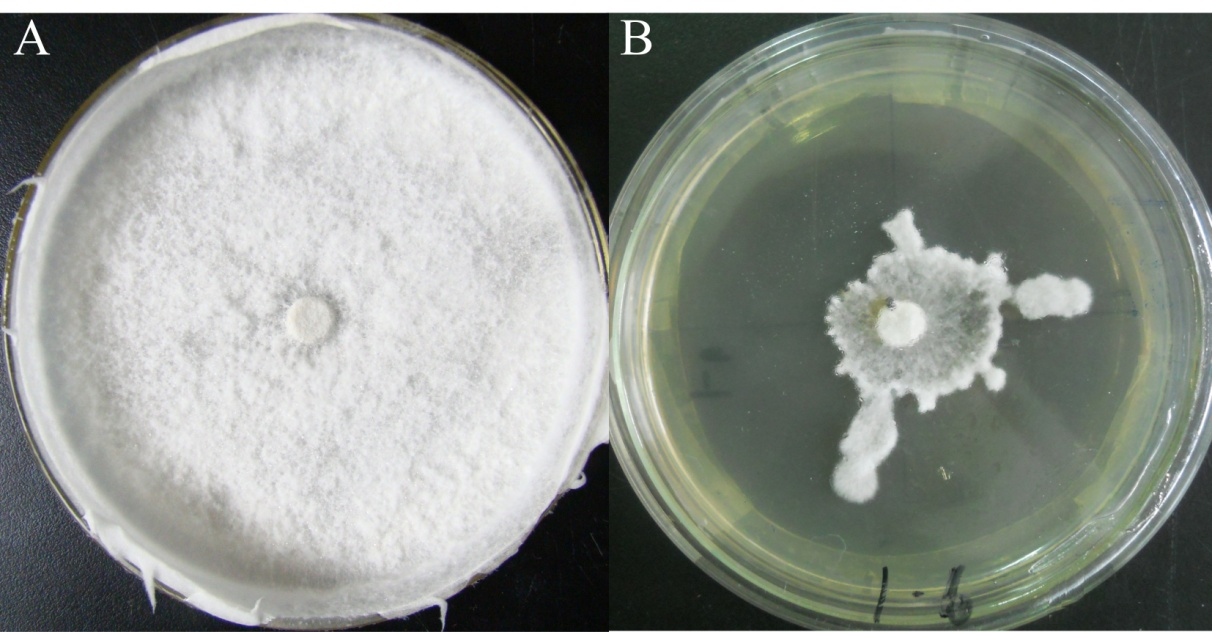

Supplement: Supplemental Information 2 [file peerj-04-1993-s002.png]

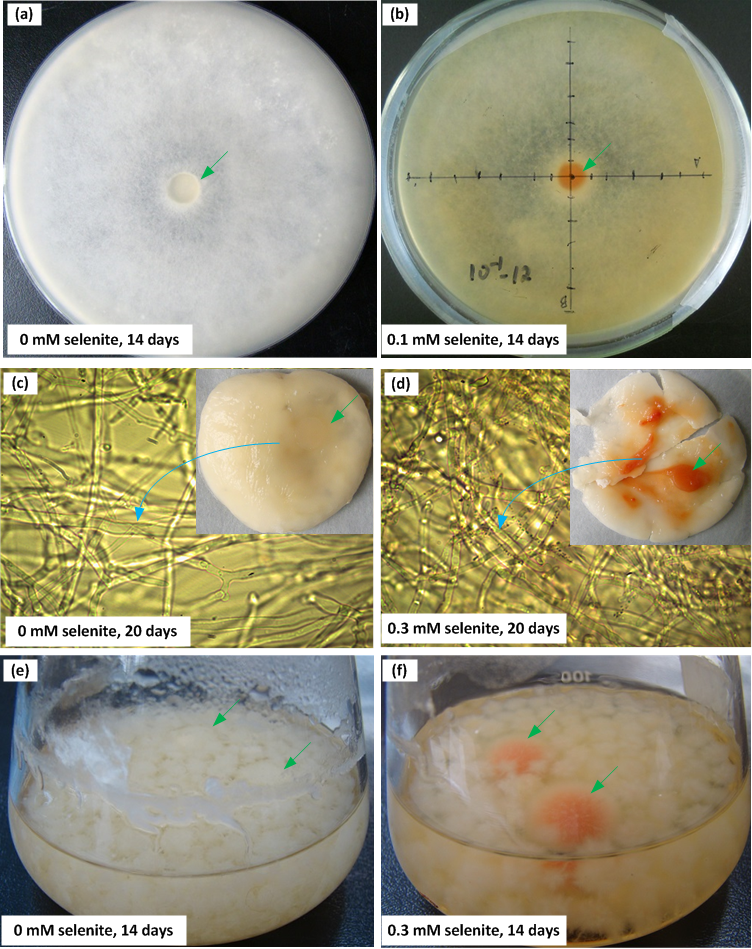

Supplement: Supplemental Information 3 — The green arrows indicate the inocula. [file peerj-04-1993-s003.png]

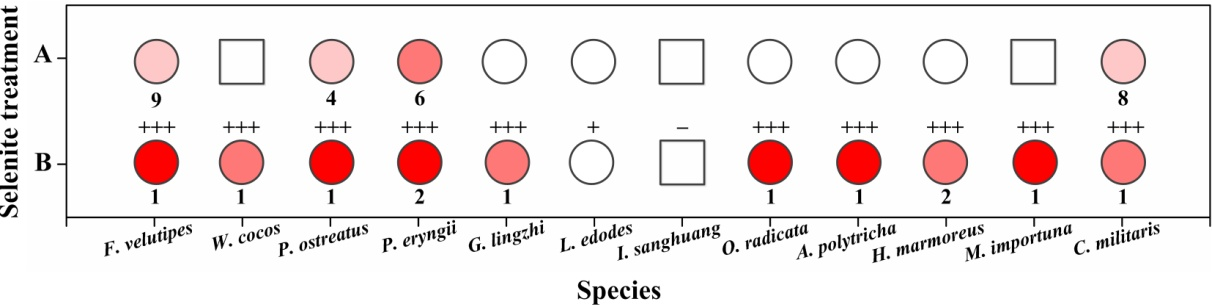

Supplement: Supplemental Information 4 — (A) solid cultivation supplied with 0.1 mM selenite; (B) shaking cultivation supplied with 0.3 mM selenite after the mycelial pellets had been cultivated in selenite-free media for 9–17 days. Intensities of the red color in the circles represent the red coloration of the colonies (only the most intense part were shown). The squares mean no clear reduction was observed because of the pigment interference. The numbers below the circles represent the time (day) when the colony started to turn red after selenite addition. The plus/minus signs on the top of circles or squares in (B) represent the intensities of garlic smell after selenite treatment for 3 days (−: none; +: low; ++: moderate; +++: high). [file peerj-04-1993-s004.png]
