# Supplementary material for: Selenium uptake, tolerance and reduction in Flammulina velutipes supplied with selenite
Source: PeerJ. 2016 May 11;4:e1993. doi: 10.7717/peerj.1993 (PMC4986802; doi:10.7717/peerj.1993)
Supplement: Supplemental Information 5 — a: All strains were provided by Soil and Fertilizer Institute, Sichuan Academy of Agricultural Sciences. [file peerj-04-1993-s005.pdf]

Table S1 The 12 species of mushrooms[a] used in this study and their ecological habits

No.	Species	Cultivar	Ecological habit
1	*Flammulina velutipes*	Chuanjin No. 11	Grows primarily on hard woods and occasionally on conifers as a white rot fungus
2	*Wolfiporia cocos*	PC1	Grows in soil (especially sandy) as a brown rot fungus and usually a parasite of *Pinus* roots
3	*Pleurotus ostreatus*	P1201	White rot decomposer of hard wood
4	*Pleurotus eryngii*	Xingbaogu-6	A weak parasite on the roots and stems of umbellifers
5	*Ganoderma lingzhi*	Chuanzhi No. 6	Grows on angiosperm (living or dead) stump, trunk and root
6	*Lentinula edodes*	L1201	Grows on trunks of the Fagaceae family as a white rot fungus
7	*Inonotus sanghuang*	SH2101	Grows on *Morus* trunks in wild
8	*Oudemansiella radicata*	O201	Grows on rotten wood, or in soil connected with buried wood by pseudorhiza
9	*Auricularia polytricha*	Chuaner No. 5	Grows on branches and decaying trunks, stumps or logs
10	*Hypsizygus marmoreus*	Lw1201	Grows on stems of maple, beech, especially on injured parts
11	*Morchella importuna*	M1S	Grows in hard wood forest, saprotrophic and symbiotic
12	*Cordyceps militaris*	C1207	A parasite of larvae or pupae of lepidopteran insects

a: All strains were provided by Soil and Fertilizer Institute, Sichuan Academy of Agricultural Sciences.
