# Supplementary material for: Selenium uptake, tolerance and reduction in Flammulina velutipes supplied with selenite
Source: PeerJ. 2016 May 11;4:e1993. doi: 10.7717/peerj.1993 (PMC4986802; doi:10.7717/peerj.1993)
Supplement: Supplemental Information 6 [file peerj-04-1993-s006.pdf]

Table S2 Phylogenetical status of the mushrooms used in this study.

No.	Species	Family	Order	Class
1	*Flammulina velutipes*	Physalacriaceae	Agaricales	Agaricomycetes
2	*Wolfiporia cocos*	Polyporaceae	Polyporales	Agaricomycetes
3	*Pleurotus ostreatus*	Pleurotaceae	Agaricales	Agaricomycetes
4	*Pleurotus eryngii*	Pleurotaceae	Agaricales	Agaricomycetes
5	*Ganoderma lingzhi*	Ganodermataceae	Polyporales	Agaricomycetes
6	*Lentinula edodes*	Marasmiaceae	Agaricales	Agaricomycetes
7	*Inonotus sanghuang*	Hymenochaetaceae	Hymenochales	Agaricomycetes
8	*Oudeman siellaradicata*	Physalacriaceae	Agaricales	Agaricomycetes
9	*Auricularia polytricha*	Auriculariaceae	Auriculariales	Agaricomycetes
10	*Hypsizygus marmoreus*	Lyophyllaceae	Agaricales	Agaricomycetes
11	*Morchella importuna*[a]	Morchellaceae	Pezizales	Pezizomycetes
12	*Cordyceps militaris*[a]	Cordycipitaceae	Hypocreales	Sordariomycetes

a: belongs to *Ascomycota*, others belong to *Basidiomycota.*
