# Supplementary material for: Selenium uptake, tolerance and reduction in Flammulina velutipes supplied with selenite
Source: PeerJ. 2016 May 11;4:e1993. doi: 10.7717/peerj.1993 (PMC4986802; doi:10.7717/peerj.1993)
Supplement: Supplemental Information 7 — The mycelial growth after the initial adaption period in the solid cultivation was fitted by a linear regression, and the growth rate was expressed as the slope k[i]. [file peerj-04-1993-s007.pdf]

Table S3 Growth rate (expressed as the slope *k*) of *F. valutipes* under selenite treatments of various concentrations and their comparisons with the selenite-free treatment.

Selenite concentration (mM)	Linear growth period (day)	*k*	*p* value
0	1 - 6	1.25	
0.001	1 - 6	1.23	0.5619
0.01	1 - 6	1.27	0.6855
0.03	1 - 6	1.25	0.8699
0.1	1 - 7	1.11	**0.0026**
0.3	1 - 7	1.10	**0.0001**
1	1 - 4	0.72	< **0.0001**

The mycelial growth after the initial adaption period in the solid cultivation was fitted by a linear regression, and the growth rate was expressed as the slope *k*.

SAS procedure for the slope comparison

SAS procedure for *k* comparison between 0 and 0.001 treatment in **Table 1**

```
data comp;
input id group time diameter@@; /*time: days after inoculation; diameter: means of colony diameter*/
cards;
1	1	1	1.025
2	1	2	2.044
3	1	3	3.311
4	1	4	4.638
5	1	5	5.908
6	1	6	7.220
7	2	1	1.045
8	2	2	1.970
9	2	3	3.235
10	2	4	4.445
11	2	5	5.839
12	2	6	7.088
;
run;
proc reg data=comp;
by group;
model diameter=time;
run;
data comp2;
set comp;
```

```
group1=.;
if group=1 then group1=1; else group1=0;
group1time=group1*time;
run;
proc reg data=comp2;
model diameter=group1 time group1time;
test group1time=0; /*if P value < 0.05, this the hypothesis is rejected, and the k values
is significantly different between 0 and 1E-3, otherwise the difference is not
significant*/
run;
```
