# Supplementary material for: Selenium uptake, tolerance and reduction in Flammulina velutipes supplied with selenite
Source: PeerJ. 2016 May 11;4:e1993. doi: 10.7717/peerj.1993 (PMC4986802; doi:10.7717/peerj.1993)
Supplement: Supplemental Information 8 — a: t-test, significance of the difference between the k[i] of selenite-free treatment and 0 (no growth was observed for the inocula after 65 days of exposure to 5 mM selenite). [file peerj-04-1993-s008.pdf]

Table S4 Growth rate (expressed as the slope *k*) of *F. valutipes* after exposing to selenite of 3 or 5 mM for 20, 23, 32 and 65 days and their comparisons with the selenite-free treatment.

Days of exposure to selinite (day)	Selenite concentraion （mM）	Linear growth period (day)	*k*	*p* value
0	0	1 - 6	1.25	
20	3	3 - 9	1.21	0.3771
	5	5 - 10	1.26	0.7772
23	3	3 - 9	1.20	0.2275
	5	4 - 9	1.06	0.0078
32	3	3 - 8	1.21	0.2640
	5	4 - 9	0.92	0.0002
65	3	2 - 8	1.14	0.1325
	5		0	<0.0001[a]

a: t-test, significance of the difference between the *k* of selenite-free treatment and 0 (no growth was observed for the inocula after 65 days of exposure to 5 mM selenite).
