# Supplementary material for: Selenium uptake, tolerance and reduction in Flammulina velutipes supplied with selenite
Source: PeerJ. 2016 May 11;4:e1993. doi: 10.7717/peerj.1993 (PMC4986802; doi:10.7717/peerj.1993)
Supplement: Supplemental Information 9 — a: The ratios were calculated based on the acid dissociation constants of H2SeO3 (K1 = 2.7 × 10^−3^, K2 = 2.5 × 10^−7^). [file peerj-04-1993-s009.pdf]

**Table S5** Ratios of different selenite species at different pH[a].

| pH | Ratio (%) | | |
|---|---|---|---|
| | $H_2SeO_3$ | $HSeO_3^{1-}$ | $SeO_3^{2-}$ |
| 5.5 | 0.111 | 92.575 | 7.313 |
| 6.5 | $6.545\times10^{-3}$ | 55.844 | 44.156 |
| 7.5 | $1.316\times10^{-4}$ | 11.227 | 88.773 |

a: The ratios were calculated based on the acid dissociation constants of $H_2SeO_3$ ($K_1=2.7\times10^{-3}$, $K_2=2.5\times10^{-7}$).
