# Supplementary material for: Selenium uptake, tolerance and reduction in Flammulina velutipes supplied with selenite
Source: PeerJ. 2016 May 11;4:e1993. doi: 10.7717/peerj.1993 (PMC4986802; doi:10.7717/peerj.1993)
Supplement: Supplemental Information 10 — a: 1–6 days of mycelial growth, b: 6–11 days of mycelial growth. c: changes of colony morphology characters other than growth rate, colony density and height, for example, shape of colony margin, appearance of sclerotium, twists of mycelia. ○: no significant change after 0.1 mM selenite treatment compared with CK, +: slight increase, ++: obvious increase, −: slight decrease, −−: obvious decrease. For growth rate, data in the parentheses are changes of k[i] after 0.1 mM selenite treatment, and those less than 10% were regarded as slight changes, those more than 10% were regarded as obvious changes. For the comprehensive sensitivity, low: no obvious change in colony characters; moderate: only one obvious change; high: two or more obvious changes. [file peerj-04-1993-s010.pdf]

Table S6 Responses of the 12 species of mushrooms to 0.1 mM selenite in the solid cultivation.

Species	Growth rate	Colony density	Colony hight	Other morphological characters[c]	Pigment secretion	Comprehensive sensitivity
F. velutipes	**− − (11.2%)**	**−**	○	○	○	**moderate**
W. cocos	− − (14.3%)	− −	− −	+ +	+ +	high
P. ostreatus	○	○	○	○	+ +	moderate
P. eryngii	○	○	○	○	○	low
G. lingzhi[a]	○	○	○	○	○	low
G. lingzhi[b]	− − (22.9%)	○	○	○	○	moderate
L. edodes	○	○	−	○	○	low
I. sanghuang	○	○	○	○	+ +	moderate
O. radicata	○	○	○	○	○	low
A. polytricha	+ (2.2%)	○	○	○	○	low
H. marmoreus	+ (8.2%)	○	○	○	○	low
M. importuna	○	−	−	+ +	+ +	high
C. militaris	+ (9.2%)	−	− −	○	−	moderate

a: 1-6 days of mycelial growth, b: 6-11 days of mycelial growth.

c: changes of colony morphology characters other than growth rate, colony density and height, for example, shape of colony margin, appearance of sclerotium, twists of mycelia.

○: no significant change after 0.1 mM selenite treatment compared with CK, +: slight increase, ++: obvious increase, -: slight decrease, --: obvious decrease. For growth rate, data in the parentheses are changes of k after 0.1 mM selenite treatment, and those less than 10% were regarded as slight changes, those more than 10% were regarded as obvious changes.

For the comprehensive sensitivity, low: no obvious change in colony characters; moderate: only one obvious change; high: two or more obvious changes.
